# Comment on “Association of kidney disease index with all‐cause and cardiovascular mortality among individuals with hypertension”

**DOI:** 10.1002/clc.24242

**Published:** 2024-02-20

**Authors:** Tomoyuki Kawada

**Affiliations:** ^1^ Department of Hygiene and Public Health Nippon Medical School Tokyo Japan

To the Editor,

I read the article by Fang et al.^1^ in “Clinical Cardiology,” who investigated the association between a novel kidney disease index (KDI), which combined information from estimated glomerular filtration rate (eGFR) and urinary albumin‐to‐creatinine ratio (uACR), and all‐cause and cardiovascular disease (CVD) mortality in patients with hypertension. The adjusted hazard ratios (95% confidence intervals) of each standard deviation increment in KDI level for all‐cause mortality and cardiovascular deaths were 1.27 (1.23–1.30) and 1.31 (1.26–1.37), respectively. Although the authors mentioned that the mortality risk increased dramatically when KDI exceeded 0.27, a significant increase was observed in patients with KDI > 0.33. I present comments with special reference to proteinuria and renal hyperfiltration.

Jonsson et al.^2^ evaluated the association between eGFR and mortality in the general population, and proteinuria was significantly associated with increased risk of death for all eGFR categories in persons of all ages. In persons ≤65 years, the lowest risk was observed for eGFR of 75–89 mL/min/1.73 m^2^ without proteinuria, and the lowest risk was observed for eGFR of 60–74 mL/min/1.73 m^2^ without proteinuria in persons >65 years. These data present that there may be age‐adapted eGFR thresholds for defining chronic kidney disease, and there may be an advantage of KDI as the combined information from eGFR and uACR for predicting mortality risk.

Jonsson et al.^2^ also clarified that eGFR >104 mL/min/1.73 m^2^ was significantly associated with increased mortality, particularly in persons >65 years. They made an emphasis that the increased mortality risk by renal hyperfiltration was observed regardless of proteinuria. Fang et al.^1^ presented that the age of subjects became higher as KDI increased. Although the prevalence of renal hyperfiltration may not be frequent, KDI would mask the pathological increase of eGFR by aging. The use of KDI should be paid attention.

Regarding hyperfiltration, increased eGFR may be derived from reduced serum creatinine levels with muscle wasting in individuals suffering from multimorbidity, which may be more observed in older subjects. Zhang et al. reported that subjects with metabolically healthy obesity presented both a significantly reduced and increased eGFR, and the association became not significant by adjusting for serum uric acid.^3^ Serum uric acid would interact with kidney function, and a mediation analysis may be one of the effective methods to evaluate the causal association between eGFR and mortality.^4^

Fang et al.^1^ analyzed data in patients with hypertension, and cardio‐renal effect on mortality would be a key point for the analysis. Taken together, I think that the advantages and masked risks of KDI may exist, which should be explored by further studies.
